# The positivity principle: do positive instructors improve learning from video lectures?

**DOI:** 10.1007/s11423-021-10057-w

**Published:** 2021-10-28

**Authors:** Alyssa P. Lawson, Richard E. Mayer, Nicoletta Adamo-Villani, Bedrich Benes, Xingyu Lei, Justin Cheng

**Affiliations:** 1 Department of Psychological and Brain Sciences, University of California, Santa Barbara, Santa Barbara, CA 93106 USA; 2 Computer Science Department, Purdue University, West Lafayette, USA

**Keywords:** Affective processes, e-learning, Emotional design, Online lesson, Video lectures

## Abstract

The positivity principle states that people learn better from instructors who display positive emotions rather than negative emotions. In two experiments, students viewed a short video lecture on a statistics topic in which an instructor stood next to a series of slides as she lectured and then they took either an immediate test (Experiment 1) or a delayed test (Experiment 2). In a between-subjects design, students saw an instructor who used her voice, body movement, gesture, facial expression, and eye gaze to display one of four emotions while lecturing: happy (positive/active), content (positive/passive), frustrated (negative/active), or bored (negative/passive). First, learners were able to recognize the emotional tone of the instructor in an instructional video lecture, particularly by more strongly rating a positive instructor as displaying positive emotions and a negative instructor as displaying negative emotions (in Experiments 1 and 2). Second, concerning building a social connection during learning, learners rated a positive instructor as more likely to facilitate learning, more credible, and more engaging than a negative instructor (in Experiments 1 and 2). Third, concerning cognitive engagement during learning, learners reported paying more attention during learning for a positive instructor than a negative instructor (in Experiments 1 and 2). Finally, concerning learning outcome, learners who had a positive instructor scored higher than learners who had a negative instructor on a delayed posttest (Experiment 2) but not an immediate posttest (Experiment 1). Overall, there is evidence for the positivity principle and the cognitive-affective model of e-learning from which it is derived.

## Objective and rationale

**Fig. 1:**
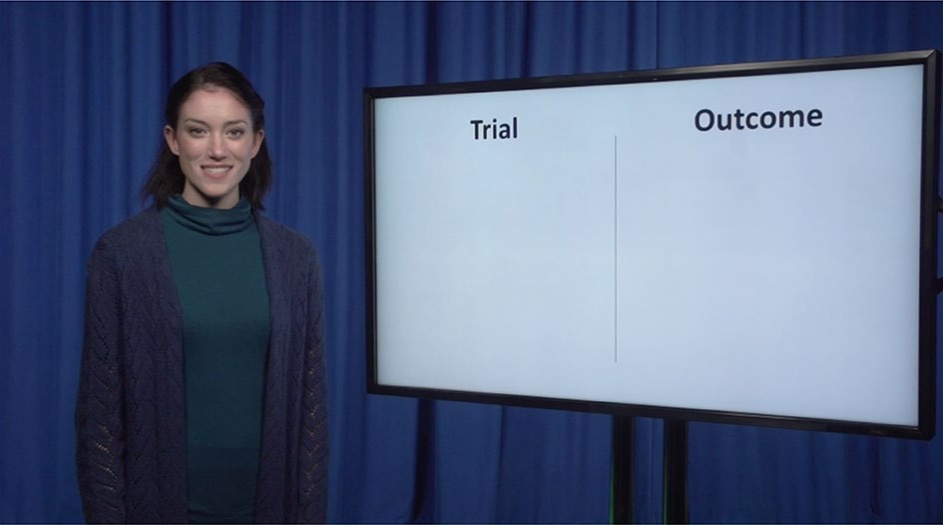
Still image from instructional video

Imagine you are watching a video lecture in which the instructor stands next to a series of slides as she explains the statistical concept of binomial probability, as shown in Fig. 1. Would you learn better if the instructor's voice, gestures, body movements, facial expression, and eye-gaze displayed positive emotions (such as seeming happy or content) rather than negative emotions (such as seeming frustrated or bored)? This is the main issue we address in the present set of experiments. A particular challenge for the design of computer-based instruction is how to elicit appropriate emotional responses in learners (Graesser et al., 2014; Plass & Kaplan, 2016; Tettegah & Gartmeier, 2016). Thus, educational technology is central to our research question because our focus is on evoking positive emotions in a computer-based learning environment.

This work is motivated by the *positivity principle* which posits that people learn better from instructors who exhibit positive emotions than from instructors who exhibit negative emotions (Lawson, et al., 2021; Mayer, 2020b). Although much work in the area of e-learning has focused on the role of cognitive factors in learning, as seen in the cognitive theory of multimedia learning (Mayer, 2020a, in press-a) or cognitive load theory (Paas & Sweller, 2014; Sweller et al., 2011), there is also a need to incorporate affective and social factors (Loderer et al., 2020; Mayer, in press-b, 2020b; Pekrun & Linnenbrink-Garcia, 2012; Pekrun & Perry, 2014) as seen in the cognitive affective model of learning with media (Moreno & Mayer, 2007) and the integrated cognitive affective model of learning with multimedia (Plass & Kaplan, 2016). Prior literature has demonstrated that emotion can play a role in cognitive load theory in various ways, including adding to extraneous load, being a beneficial or harmful factor in memory depending on the type of emotion felt, creating an additional component to process while learning, and serving as a motivational factor (Fraser et al., 2014; Knörzer et al., 2016; Plass & Kalyuga, 2019). This project represents an attempt to continue to take on this challenge by focusing specifically on how the impact of displayed emotions by an instructor during a video lecture can be incorporated into cognitive theories to provide a more well-rounded understanding of computer-based learning.

The role of emotion in academic learning is well-founded in the literature, which indicates that emotions are an important element to consider in teaching and learning (e.g., Becker et al., 2014; Brünken et al., 2010; Christianson, 1992; Knörzer et al., 2016; Pekrun, 2011, 2017; Pekrun et al., 2011; Plass & Kalyuga, 2019; Tyng et al., 2017). It is important to distinguish between the learner's felt emotion during learning and the instructor's portrayed emotion during instruction; in this study we focus on the impact of the instructor's portrayed emotion in an instructional video as our independent variable. We focus on instructional video because it is an increasingly important venue for instruction, including its use in flipped classrooms as a resource in class management systems, online instruction, and MOOCs (Bonk et al., 2015; Derry et al., 2014; Fiorella & Mayer, 2018; Mayer et al., 2020). With video lectures, social and affective factors come into play, including the instructor's gestures, movements, and eye-gaze (Fiorella et al., 2019, 2020). The growing research base on instructional video contributes to the broader field of e-learning (Fiorella, in press; Mayer et al., 2020), and this project represents an initiative to contribute to what we know about how to design instructional video to improve student learning. Particularly, we aim to investigate how the emotions displayed by an instructor may impact learning, specifically using instructional videos.

When an instructor is teaching a lesson online, it is essential to understand which aspects of that video a student may attend to and how that influences learning. Much research has investigated how different aspects of a lesson can influence student learning, such as spacing, gesturing, pointing, etc. (Mayer, 2020a, in press-a). One aspect of an instructor’s presentation of material that has not been studied as much in the context of having a direct effect on learning is the instructor’s emotional tone. Much research supports that learners respond to the emotions of an instructors in person and through e-learning (e.g., Becker et al., 2014; Fiorella, in press; Rowe et al., 2013; Saneiro et al., 2014), yet there has been less research to investigate how the instructor's emotion plays a role in learning from an online video lesson (Fiorella, in press; Mayer et al., 2020). Although the learner's cultural background can affect how they interpret the instructor's displayed emotion (e.g., Engelmann & Pogosyan, 2013; Fang et al., 2017; Grossmann et al., 2011), we did not address the cultural aspects of emotional design in this study.

This set of studies investigates how an instructor’s emotions may play a role in student learning. In order to investigate this, four videos were created on the same material about binomial probability only differing in the emotion that the instructor displayed (i.e., happy, content, frustrated, or bored). Students watched one of the four videos and then took a posttest on the material covered in the video. If an instructor’s emotion does play a role in how students learn from a lesson, this should be reflected in each group’s performance on the posttest. For instance, if positive emotions (such as happy or content) lead to better learning than negative emotions (such as frustrated or bored), students who see an instructor displaying a positive emotion should perform better on the posttest than those who see an instructor displaying a negative emotion.

## Literature review

**Fig. 2:**
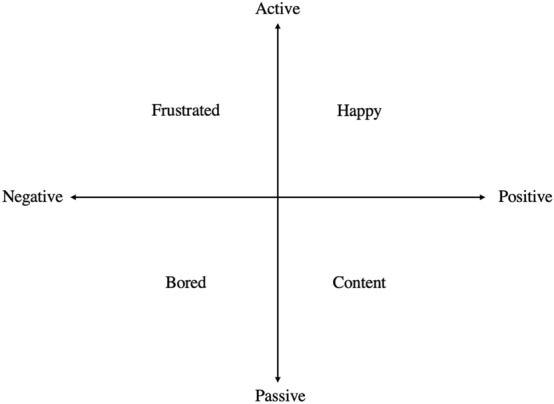
Adapted version of Russell’s (1980, 2003) model of core affect

This work is motivated by Russell’s (1980, 2003) model of core affect, although we use slightly different terminology. Figure 2 shows an adapted version of Russell’s (1980, 2003) model displaying two orthogonal dimensions: valence (which runs from positive to negative) and arousal, or what we have called activity (which runs from active to passive). We prefer to use the term *activity* because we are focusing on the instructor's portrayed emotion, although we adhere to Russell's framework. These dimensions create four quadrants, similar to Pekrun and Perry’s (2014; Loderer et al., 2019) taxonomy of achievement motivation, which we have represented as *happy* (positive/active), *content* (positive/passive), *frustrated* (negative/active), and *bored* (negative/passive). These are the four emotions displayed by instructors in our study.

The present study also builds on the emerging research base on what has been called *emotional design*, i.e., examining the role of instructional design features that convey emotion (Loderer et al., 2020; Mayer, 2020b; Pawar et al., 2019; Plass & Kaplan, 2016). In a set of groundbreaking studies, Plass et al. (2014) and Um et al. (2012) added emotional design components into a computer-based lesson on immunization in order to understand how learners react. In both studies, students learned better when the characters in the lesson were more emotionally appealing, i.e., displayed in warm colors (rather than gray) and with rounded faces and bodies (rather than square ones).

Mayer and Estrella (2014) found similar results in their study on teaching learners about viruses via a slideshow. In the control condition, the main characters (e.g., the virus and the host cell) were illustrated as black-and-white line drawings without any facial features; whereas in the emotional design condition, the main characters were presented in warm colors with facial expressions. As in the foregoing studies, students performed better on learning outcome tests when emotional design features were added to the computer-based lesson.

Prior literature has provided support for the viewpoint that emotions play a role in cognition. In a recent review, Loderer et al. (2020) found a relation between the emotions experienced during e-learning and the learner’s level of cognitive processing during e-learning on the learner’s performance on tests of learning outcome. Duffy et al. (2020) also reported a positive relation between learners’ experiencing of positive emotions during learning and their performance on tests of learning outcome. Plass et al. (2020) found that game players rated in-game characters as happier if they were displayed with warm colors and positive facial expressions. However, some literature posits that some emotions can negatively impact learning, as these emotions add to extraneous load that a learner experiences (Fraser, et al., 2014; Knörzer et al., 2016; Plass & Kalyuga, 2019). For example, Knörzer et al. (2016) induced either a positive, neutral, or negative emotion in their learners prior to learning. Then, participants learned a biology lesson using text and pictures. Those who were induced with positive emotions did significantly worse on a posttest assessing comprehension and transfer than those who were inducted with negative emotions.

Studies like these help illuminate how emotions may play an important role in learning, mainly focusing on how emotional design influences learning. The previous research focuses on the learner’s emotion and the impact this emotion can play in the cognitive experience of learning. The current study expands this previous research by focusing on how the emotions displayed by an instructor in a video lecture influences a learner’s affective and cognitive processing during learning as well as outcome performance. Affective processing refers to the learner recognizing the emotional tone of the instructor and adopting their own emotional tone during the lesson; whereas cognitive processing refers to internal mental activity such as attending to incoming information, mentally organizing it into a coherent structure, and relating it to relevant prior knowledge (Mayer, 2020a).

**Fig. 3:**
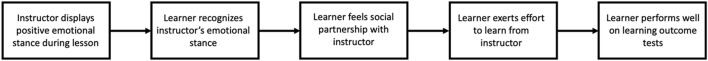
Cognitive affective model of e-learning

In particular, this study investigates the cognitive affective model of e-learning, displayed in Fig. 3 (Lawson et al., 2021; Mayer, 2020b). In this model, there are 4 steps that lead from the emotion displayed by an instructor to an effect on learning outcome. First, instructors must display a positive emotion during a lesson. This leads to the learner recognizing the instructor’s emotional stance (step 1) which then leads to the learner feeling a social partnership with the instructor (step 2). Once that partnership is established, the learner will work hard to learn the lesson (step 3) and thus perform better on learning outcome tests (step 4). We use this model to inform how we conducted these experiments.

We acknowledge that the affective interactions between student and teacher are more complex than the linear model presented in Fig. 3 (Loderer et al., 2020; Pekrun & Linnenbrink-Garcia, 2012; Pekrun & Perry, 2014), so this study can be seen as a preliminary step in establishing basic relations, focusing on the role of an instructor’s emotion in learning. In addition, the subject matter (e.g., statistics) and learning context (e.g., computer-based instruction), which were not varied in this study, may elicit emotional responses in the learner independent of the instructor (e.g., displaying positive or negative emotion), which was varied in this study (Graesser et al., 2014; Tettegah & Gartmeier, 2016); thus, further work is needed to disentangle the emotional response to a computer-based statistics lesson.

## Theory and predictions

The positivity principle explains that people learn better from instructors who display a positive emotional tone rather than from instructors who display a negative emotional tone. The positivity principle is based on a cognitive-affective model of e-learning summarized in Fig. 3 (Lawson et al., 2021; Mayer, 2020b). The model consists of four crucial links, each yielding a hypothesis for testing the positivity principle within the context of learning from a video lecture. The chain of events begins when the learner receives a video lecture in which an instructor who exhibits a positive or negative emotional tone stands next to a PowerPoint slide as she lectures. The model represents a set of to-be-tested steps concerning the role of emotion in learning from instructional videos, rather than an established set of findings.

The first proposed step is that the learner recognizes the emotional tone being displayed by the instructor, that is, the learner recognizes that a positive instructor is displaying a positive emotion and a negative instructor is displaying a negative emotion. According to the model, this is an essential first step (Lawson et al., 2021). The second step is that the emotional tone of the instructor affects social processing in the learner, that is, the learner feels a stronger social connection with the positive instructor than with the negative instructor. The third step is that the emotional tone of the instructor affects cognitive processing in the learner, that is, the learner works harder to learn the material with a positive instructor than with a negative instructor. The final–and most educationally important step–is that the emotional tone of the instructor affects the learning outcome, that is, the learner builds a better understanding of the material with a positive instructor than with a negative instructor. This statement reflects a to-be-tested research hypothesis rather than an established fact, but is consistent with much of the research base on emotional design (Mayer & Estrella, 2014; Mayer, 2020a; Plass & Kalyuga, 2019; Plass & Kaplan, 2016; Plass et al., 2014; Um et al., 2012).

This analysis leads to four specific predictions which we tested in the present experiments. First, concerning recognizing the emotional tone of the instructor, learners who view a video lecture with a positive instructor (i.e., an instructor who displays happy or content emotion) will rate the instructor's emotion higher on positive emotions whereas learners who view a video lecture with a negative instructor (i.e., an instructor who displays frustrated or bored emotion) will rate the instructor’s emotion higher on negative emotions (hypothesis 1). This means that participants who see the happy instructor will rate the instructor as happier than each of the other three emotions (hypothesis 1a); participants who see the content instructor will rate the instructor as more content than each of the other three conditions (hypothesis 1b); participants who see the bored instructor will rate the instructor as more bored than each of the other three conditions (hypothesis 1c); and participants who see the frustrated instructor will rate the instructor as more frustrated than each of the other three conditions (hypothesis 1d). In step 2 of the model, participants who have a positive instructor will have higher ratings of social connection with the instructor including higher ratings of facilitating learning, credibility, and engagement than participants who have a negative instructor (hypothesis 2). In step 3 of the model, participants who have a positive instructor will report working harder to understand the material than participants who have a negative instructor (hypothesis 3). Given the preliminary nature of our self-report motivational measures, we consider this to be an exploratory issue in this study. Lastly, based on the fourth step of the model, participants who have a positive instructor will have better scores on a learning outcome posttest than participants who have a negative instructor (hypothesis 4).

## Experiment 1

### Method

#### Participants and design

The participants were 96 students recruited from a psychology subject pool at a university in southern California. Their mean age was 19.56 years (*SD* = 1.42); 62 were women, 33 were men, and one did not indicate a gender. The experiment used a 2 (valence of emotion: positive or negative) × 2 (activity of emotion: active or passive) between-subjects design. This created 4 groups: 23 participants served in the happy instructor (positive/active) group, 25 participants were in the content instructor (positive/passive) group, 24 participants were in the frustrated instructor (negative/active) group, and 24 participants were in the bored instructor (negative/passive) group.

#### Materials

The paper-based materials consisted of a prequestionnaire and a postquestionnaire. The computer-based materials consisted of 4 versions of an instructional video on binomial probability and a posttest consisting of 21 questions that were presented in a self-paced series of slides.

##### Prequestionnaire

The prequestionnaire solicited demographic information from students (including their age and gender); it also included a list of math classes the students were asked to mark if and when they had each class (e.g., pre-algebra, algebra, etc.); and finally, it had a statement asking students to rate their knowledge of statistics on a 5-point scale from “very low” to “very high,” and included a list of 11 statements relating to statistics along with instructions for students to place a checkmark next to each statement that applied to them (e.g., “I have taken a statistics class,” “I remember learning about probability in math class,” and “I know the difference between combinations and permutations”). The score from the scale and the number of checks the students had marked were added together to make a composite score of prior knowledge. The Cronbach’s alpha for prior knowledge items was .63. The internal validity for this measure is likely low due to the fact that the prequestionnaire attempted to gain an understanding of the general knowledge or familiarity that participants had of concepts related to binomial probability and statistics, rather than a single concept. The items were selected based on recommendations for how to measure familiarity with the topic, without creating a testing effect or priming effect in which taking a pretest is a learning episode that also guides the learner's attention during the lesson (Mayer, 2020a).

##### Video lessons

The instructional materials consisted of four versions of a recorded video lesson in which a young woman instructor stood next to a series of slides as she explained the statistical topic of binomial probability. A screenshot is shown in Fig. 1. The script is provided in Appendix A and was adapted from an earlier paper-based lesson developed by Mayer and Greeno (1972). The lesson lasted approximately 10 min (depending on the emotional tone of the instructor). All versions of the lesson had the same instructor using the same script and slides to explain binomial probability.

The four versions of the video lecture were based on the emotional tone displayed by the instructor: happy (positive/active), content (positive/passive), frustrated (negative/active), or bored (negative/passive). These emotions were displayed through the instructor's voice, gestures, facial expression, body positioning, and eye gaze. In the happy video, the instructor had an upbeat voice while her body was open and leaning forward. In the content video, the instructor had a calmer voice while her body was open and leaning backwards. In the frustrated video, the instructor had an annoyed voice while her body was closed and leaning forward. In the bored video, the instructor had a monotone voice while her body was closed and leaning backwards.

The instructor—a young woman in casual attire–was a student actor from the university's Theater Department. The four versions of the lecture were recorded at a professional recording studio in the university's Instructional Development Department. Two experimenters attended all recording sessions to make sure the script was followed perfectly and that the instructor was displaying the appropriate emotional tone. The actor was given written and oral instructions for body stance, gestures, and voice to be used for each emotion (as summarized above) and received feedback from the two experimenters during rehearsal until the expected emotional tone was achieved. Also, during production, when necessary, an experimenter called for retakes of portions of the lesson. The four versions were produced as mp4 files that could run locally on iMac computers.

##### Posttest

The posttest consisted of 21 items, each presented on a separate PowerPoint slide. These included questions asking students to recall the definitions of the different symbols from the equations (“What does N symbolize?”), solve problems using formulas (“P = 1/2, N = 8, R = 5, What is C(N,R)?”), recognize unsolvable problems (“N = 2, R = 3, P = 1/2, What is C(N,R)?”), solve word problems (“Is there a difference between the probability that two dice rolled at once both come up 6 and the probability that one die rolled twice comes up 6 both times?”) and answer questions (“Can P be greater than 1−P?”). The posttest was presented as a PowerPoint slideshow, and participants could advance to the next slide at their own pace through the posttest. Cronbach’s alpha for the posttest was .79. A reason for the low internal consistency of this exam is that the test consisted of various types of questions in order to understand participants’ understanding of the presented material. These question types ranged from definitional questions to recognizing impossible situations. We constructed five types of problems based on previous research by Mayer and Greeno (1972), including rote memory items, solving computational problems stated in formula format, solving computational problems stated as word problems, answering conceptual questions, and recognizing unanswerable problems.

##### Postquestionnaire

The postquestionnaire included individual questions that were intended to assess the learner’s experience with the lesson. First, participants were asked to rate the degree to which the instructor displayed each of the four emotions (happy, content, frustrated, and bored) on a 5-point scale from "strongly disagree" to "strongly agree". These four items were intended to help determine how well participants were able to recognize the emotion being displayed by the instructor they saw. Second, participants were asked to use a 5-point scale to rate their level of paying attention, effort, and enjoyment, as well as how difficult the material was and how much they would like to receive more lessons like the one they saw. These items are intended to provide preliminary information about the cognitive processing (i.e., paying attention, exerting effort) and affective processing (i.e., enjoyment, difficulty, and wanting more lessons). Third, the postquestionnaire contained 10 items asking the participant to rate how well the instructor facilitated learning (Cronbach's alpha =  .93), 4 items on how credible the instructor was (Cronbach's alpha =  .92), and 5 items on how engaging the instructor was (Cronbach's alpha = .84). These items were taken from the Agent Persona Instrument (API; Baylor & Ryu, 2003).

#### Apparatus

The apparatus consisted of 4 iMac computer systems, with 20-inch color screens and over-the-ear headphones, each housed in an individual cubicle that blocked visual contact among participants.

#### Procedure

Participants were randomly assigned to one of the four conditions and tested in individual cubicles in a lab setting with up to four participants in each session. All participants in the same session were randomly assigned to the same group. First, participants received an overview of the study and signed the informed consent form. Next, participants completed the prequestionnaire at their own pace. Next, the experimenter provided oral instructions on how to complete the experiment, and the participants began watching the video lesson on an iMac computer. Following the lesson, participants took an immediate posttest as a series of slides on an iMac computer. The posttest consisted of 21 questions related to binomial probability. Participants were allowed to work through the posttest one item at a time and move on to the next item at their own pace. They were given a simple calculator to help with calculations. They wrote their answers on several pre-numbered sheets of paper. Participants took an average of 26 min to complete the posttest. Once the participants finished the posttest, they were given the postquestionnaire to complete at their own pace. Participants were given time to complete the lesson, posttest, and postquestionnaire at their own pace in order to allow for those that needed more processing time to do so, in line with research showing the benefits of a power tests rather than speed tests for under-represented groups (Zwick, 2002). The entire experiment took up to an hour. We obtained IRB approval and adhered to guidelines for ethical treatment of human subjects.

### Results

#### Do the groups differ on basic characteristics?

A preliminary issue concerns whether random assignment produced groups that were equivalent on basic characteristics. Concerning prior knowledge score, there were no statistically significant differences between the groups based on valence, *F*(1, 99) = .004, *p* = .953, nor based on activity, *F*(1, 99) = .15, *p* = .700, and no significant interaction, *F*(1, 99) = .34, *p* = .563. Concerning age, there were no statistically significant differences between the groups based on valence, *F*(1, 99) = .24, *p* = .623, nor based on activity, *F*(1, 99) = .56, *p* = .457, and no significant interaction, *F*(1, 99) = 2.48, *p* = .119. Concerning number of prior math classes taken, there were no statistically significant differences between the groups based on valence, *F*(1, 99) = .69, *p* = .407, nor based on activity, *F*(1, 99) = 1.05, *p* = .308, and no interaction, *F*(1, 99) = .06, *p* = .802. Concerning gender, a chi-square test showed that there were no statistically significant differences among the groups, χ^2^(3, *N* = 102) = 3.78, *p* = .286. Based on the fact that there are no significant differences in any of the statistical tests among the groups, we conclude that participants in each group were equivalent in the basic characteristics of prior knowledge, age, number of prior math courses, and gender composition.

#### Hypothesis 1: Do students recognize whether an instructor is displaying positive or negative emotions?

From the cognitive affective model of e-learning shown in Fig. 3, the first step is for learners to recognize the emotions displayed by the instructor. In particular, based on the positivity principle, we are interested in whether learners give more positive emotional ratings when the instructor displayed positive emotions than when the instructor displayed negative emotions (hypothesis 1). To address this issue, the data was organized into four sets, one for each emotion (happy, content, frustration, and bored). Rating means and standard deviations are reported in Table 1. For each rated emotion, we conducted 2 × 2 ANOVAs with the factors being valence (positive versus negative) and activity (active versus passive), followed up with one-way ANOVAs.

**Table 1:** Means and standard deviations of emotional ratings of the 4 video lessons in Experiment 1

	Happy rating		Content rating		Bored rating		Frustrated rating	
	*M*	*SD*	*M*	*SD*	*M*	*SD*	*M*	*SD*
Happy video	**4.38**	.75	4.23	.71	1.50*	.86	1.35*	.75
Content video	3.62*	.75	**3.92**	.74	2.42*	.95	1.65*	.75
Bored video	1.36*	.91	1.56*	1.00	4.44*	1.23	3.92	1.19
Frustrated video	1.31*	.68	1.65*	.98	4.58	.58	**3.92**	1.32

Asterisk(*) represents significant difference from the target emotion

First, concerning ratings of how happy the instructor was, there was a significant effect of valence favoring the positivity principle in which participants who saw the instructor displaying positive emotions (*M* = 4.00, *SD* = .84) gave a higher happy rating than those who saw the instructor displaying negative emotions (*M* = 1.33, *SD* = .79), *F*(1, 99) = 303.93, *p* < .001, *d* = 3.25. There also was a significant effect of activity, in which participants who the instructor displaying the active emotions (*M* = 2.85, *SD* = 1.71) gave a higher happy rating than those who saw the instructor displaying passive emotions (*M* = 2.51, *SD* = 1.41), *F*(1, 99) = 5.50, *p* = .021, *d* = .22. Lastly, there was a significant interaction, *F*(1, 99) = 7.21, *p* = .008. As a follow-up, we conducted a one-way ANOVA for differences in the rating of happy emotion across the four groups. There was a significant difference in ratings of happy emotion among conditions, *F*(3, 99) = 105.65, *p* < .001. Post-hoc Dunnett’s tests (with p < .05) showed that the happy group gave a significantly higher happy rating than the content, bored, and frustrated groups. Consistent with hypothesis 1a of the positivity principle, learners were able to tell the difference between the level of happiness when the instructor displayed positive versus negative emotions.

Second, concerning ratings of how content the instructor was, there was a significant effect of valence in which participants who saw the instructor displaying positive emotions (*M* = 4.08, *SD* = .74) gave a higher content rating than those who saw the instructor displaying negative emotions (*M* = 1.61, *SD* = .98), *F*(1, 99) = 208.68, *p* < .001, *d* = 2.85 There was not a significant effect of activity, *F*(1, 99) = 1.38, *p* = .243, nor a significant interaction, *F*(1, 99) = .39, *p* = .533. As a follow-up, we conducted a one-way ANOVA for differences in the rating of content emotion across the four groups. There was a significant difference in ratings of content emotion among conditions, *F*(3, 99) = 70.12, *p* < .001. Post-hoc Dunnett’s tests (with p < .05) showed that the content group gave a significantly higher content rating than the bored and frustrated groups but did not differ significantly from the happy group. Consistent with hypothesis 1b of the positivity principle, learners were able to tell the difference between the level of contentment when the instructor displayed positive versus negative emotions..

Third, concerning ratings of how bored the instructor was, there was a significant effect of valence in which participants who saw the instructor displaying negative emotions (*M* = 4.51, *SD* = .95) gave a higher bored rating than those who saw the instructor displaying positive emotions (*M* = 1.96, *SD* = 1.01), *F*(1, 99) = 193.69, *p* < .001, *d* = 2.61. There was a significant effect of activity in which participants who saw the instructor displaying passive emotions (*M* = 3.41, *SD* = 1.49) gave a higher bored rating than those who saw the instructor displaying active emotions (*M* = 3.04, *SD* = 1.72*), F*(1, 99) = 4.61*, p* = .034*, d* = .23. Lastly, there was a significant interaction, *F*(1, 99) = 8.39, *p* = .005. As a follow-up, we conducted a one-way ANOVA for differences in the rating of bored emotion across the four groups. There was a significant difference in ratings of the bored emotion among conditions, *F*(3, 99) = 69.02, *p* < .001. Post-hoc Dunnett’s tests (with p < .05) showed that the bored group gave a significantly higher bored rating than the happy and content groups but did not differ significantly from the frustrated group. Consistent with hypothesis 1c of the positivity principle, learners were able to tell the difference between the level of boredom when the instructor displayed positive versus negative emotions.

Fourth, concerning ratings of how frustrated the instructor was, there was a significant effect of valence in which participants who saw the instructor display negative emotions (*M* = 3.92, *SD* = 1.25) gave a higher frustrated rating than those who saw the instructor display positive emotions (*M* = 1.50, *SD* = .75), *F*(1, 99) = 141.71, *p* < .001, *d* = 2.35 There was not a significant effect of activity, *F*(1, 99) = .56, *p* = .456, nor a significant interaction, *F*(1, 99) = .58, *p* = .447. As a follow-up, we conducted a one-way ANOVA for differences in the rating of frustrated emotion across the four groups. There was a significant difference in ratings of frustrated emotion among conditions, *F*(1, 99) = 47.63, *p* < .001. Post-hoc Dunnett’s tests (with p < .05) showed that the frustrated group gave a significantly higher frustrated rating than the happy and content groups but did not differ significantly from the bored group. Consistent with hypothesis 1d of the positivity principle, learners were able to tell the difference between the level of frustration when the instructor displayed positive versus negative emotions.

Overall, there is consistent evidence for the first hypothesis of the positivity principle: students were able to distinguish between the instructor’s positive and negative emotional tones. In addition, there was partial evidence that students were able distinguish the instructor’s active and passive emotional tones in some situations but not others.

#### Hypothesis 2: Do students feel more social connection with positive instructors?

The next step in the cognitive affective model of e-learning shown in Fig. 3 is that once the learner recognizes the emotions of the instructor, this should influence the learner’s perception of the instructor as a valued social partner. To assess this idea, the participants gave ratings about three features of the instructor based on items from the Agent Persona Instrument (API; Baylor, & Ryu, 2003). Mean ratings and standard deviations for each group on each of the factors are reported in Table 2.

**Table 2:** Means and standard deviations for three subscales of the API in Experiment 1

API factors	Positive/active (happy)		Positive/passive (content)		Negative/passive (bored)		Negative/active (frustrated)	
	*M*	*SD*	*M*	*SD*	*M*	*SD*	*M*	*SD*
Facilitating learning	3.43	.63	2.76	.71	1.63	.65	1.81	.62
Credible	3.97	.70	3.82	.60	2.65	.92	3.08	.87
Engaging	3.93	.61	2.82	.87	1.32	.40	1.69	.77

As shown in the first row of Table 2, the first factor is the ability of the instructor to facilitate learning. Consistent with the positivity principle, there was a significant effect of valence, *F*(1, 98) = 113.52, *p* < .001, *d* = 2.00, with participants rating the instructor displaying positive emotions (*M* = 3.10, *SD* = .74) as better at facilitating learning compared to the instructor displaying negative emotions (*M* = 1.72, *SD* = .63). There was also a significant effect of activity, *F*(1, 98) = 10.91, *p* = .001, *d* = .45, with the instructor displaying active emotions (*M* = 2.64, *SD* = 1.02) rated as better at facilitating learning than the instructor displaying passive emotions (*M* = 2.21, *SD* = .88). Finally, there was not a significant interaction, *F*(1, 98) = 3.53, *p* = .063. The valence and activity of the instructor’s emotion were important to how participants perceived the instructor’s ability to facilitate learning, consistent with hypothesis 2.

The second factor from the API is credibility. Consistent with the positivity principle, there was a significant effect of valence, *F*(1, 98) = 44.22, *p* < .001, *d* = 1.29 with participants rating the instructor displaying positive emotions (*M* = 3.89, *SD* = .65) as more credible than the instructor displaying negative emotions (*M* = 2.87, *SD* = .91). There was no significant effect of activity, *F*(1, 98) = 3.50, *p* = .064, and there was no significant interaction, *F*(1, 98) = .78, *p* = .378. The emotional valence of the instructor was important in how participants perceived the instructor’s credibility, again consistent with hypothesis 2.

Lastly, the third factor from the API is the degree to which the instructor is engaging. In line with the positivity principle, there was a significant effect of valence, *F*(1, 98) = 188.00, *p* < .001, *d* = 2.32 with the instructor displaying positive emotions (*M* = 3.36, *SD* = .93) being rated as more engaging than the instructor displaying negative emotions (*M* = 1.51, *SD* = .64). There was also a significant effect of activity, *F*(1, 98) = 29.78, *p* < .001, *d* = .60, with the instructor displaying active emotions (*M* = 2.79, *SD* = 1.32) being rated as more engaging than instructor displaying passive emotions (*M* = 2.08, *SD* = 1.01). There was also a significant interaction, *F*(1, 98) = 7.40, *p* = .008. The interaction revealed that when the instructor was positive, she was rated as significantly more engaging when also active (happy; *M* = 3.93, *SD* = .61) than when she was also passive (content; *M* = 2.82, *SD* = .87), *t*(44.93) = 5.33, *p* < .001; and, for when the instructor was negative, she was rated as significantly more engaging when also active (frustrated; *M* = 1.69, *SD* = .772) than when she was also passive (bored; *M* = 1.32, *SD* = .396), but by a smaller margin *t*(37.61) = 2.18, *p* = .036. We conclude that the instructor’s emotion affected participants' perceptions of how engaging the instructor was, again consistent with hypothesis 2.

Overall, the results consistently support the second step in the positivity principle (hypothesis 2) in that when the instructor has positive emotions, she was rated higher in facilitating learning, more credible, and more engaging than when she displayed negative emotions. In addition, there is partial support for what can be called the enthusiasm principle, in that when the instructor had active emotions, she was rated higher in facilitating learning and more engaging than when she had passive emotions. Overall, in line with hypothesis 2, the emotion of the instructor affected the learner’s perceptions of important aspects of the instructor as a valued social partner.

#### Hypothesis 3: Do students try harder to learn with positive instructors?

The third step in the cognitive affective model of e-learning is that if the learner perceives the instructor as more positive, the learner should work harder to try to learn the material. Means and standard deviations for each postquestionnaire question on cognitive processing during learning are reported in Table 3. In order to understand if the participants worked harder in learning the material due to the instructor’s emotion, 2 × 2 ANOVAs were run on each postquestionnaire question. We used item-level analysis in light of the preliminary nature of our measures. Each of the rating items assesses a different aspect of the learner's experience, and we did not have a conceptual justification for compiling them into a composite.

**Table 3:** Means and standard deviations for postquestionnaire questions in Experiment 1

Questionnaire items	Positive/active (happy)		Positive/passive (content)		Negative/passive (bored)		Negative/active (frustrated)	
	*M*	*SD*	*M*	*SD*	*M*	*SD*	*M*	*SD*
Pay attention	3.19	1.27	2.73	1.51	1.72	.98	1.96	.82
Difficulty	2.77	.99	2.77	1.28	2.16	.99	2.81	1.06
Effort	2.77	.99	2.81	.98	2.64	1.00	2.62	1.02
Enjoy	2.08	.98	2.15	1.08	2.24	1.30	2.38	.98
More lessons	2.12	.95	1.96	1.08	1.72	1.17	2.04	1.22

The first question analyzed was “I was motivated to pay attention to the lesson I just watched.” There was a significant effect of valence, *F*(1, 99) = 28.29, *p* < .001, *d* = 1.07, with participants indicating they were more motivated to pay attention when the instructor displayed positive emotions (*M* = 2.96, *SD* = 1.22) compared to when she displayed negative emotions (*M* = 1.84, *SD* = .903), consistent with hypothesis 3. There was no effect of activity, *F*(1, 99) = 2.78, *p* = .098, nor an interaction, *F*(1, 99) = .27, *p* = .603. In line with hypothesis 3 of the positivity principle, participants tried to pay attention more when the instructor was positive than when the instructor was negative.

The second question was “I put a lot of effort to understand the information in the lesson.” There was no effect of valence, *F*(1, 99) = .67, *p* = .416, no effect of activity, *F*(1, 99) = .03, *p* = .873, nor an interaction, *F*(1, 99) = .001, *p* = .972, inconsistent with hypothesis 3.

The third question was “The information in the lesson was difficult for me.” There was no effect of valence, *F*(1, 99) = 1.78, *p* = .185, no effect of activity, *F*(1, 99) = 2.29, *p* = .133, nor an interaction, *F*(1, 99) = 2.29, *p* = .133, inconsistent with hypothesis 3.

The fourth question was “I enjoyed learning about this information.” There was no effect of valence, *F*(1, 99) = .84, *p* = .362, no effect of activity, *F*(1, 99) = .03, *p* = .875, nor an interaction, *F*(1, 99) = .27, *p* = .608, inconsistent with hypothesis 3.

The fifth question was “I would like more lessons like this one.” There was no effect of valence, *F*(1, 99) = .53, *p* = .468, no effect of activity, *F*(1, 99) = 1.17, *p* = .282, nor an interaction, *F*(1, 99) = .14, *p* = .707, inconsistent with hypothesis 3.

Overall, there is some support based on one of five subjective self-report learning measures that the instructor’s level of positivity influenced the learner’s willingness to try hard to understand the lesson. However, ratings on four out of five measures were not consistent with hypothesis 3.

#### Hypothesis 4: Do students learn better from positive instructors?

The last step in the cognitive affective model of e-learning is that students who were taught by a positive instructor should perform better on posttests than students who were taught by a negative instructor. Means and standard deviations on the posttest for each group are reported in Table 4. A 2 × 2 ANOVA showed there was not a significant effect of valence, *F*(1, 99) = .11, *p* = .738, not a significant effect of activity, *F*(1, 99) = .61, *p* = .438, and not a significant interaction, *F*(1, 99) = .08, *p* = .772, inconsistent with hypothesis 4. The instructor’s emotion was not a significant factor in participants’ performance on an immediate posttest.

**Table 4:** Means and standard deviations on posttest in Experiment 1

	Positive/active (happy)		Positive/passive (content)		Negative/passive (bored)		Negative/active (frustrated)	
	*M*	*SD*	*M*	*SD*	*M*	*SD*	*M*	*SD*
Posttest	.53	.20	.55	.21	.55	.20	.51	.19

### Discussion

This experiment found evidence for the first two links in the positivity principle. First, concerning recognition of the instructor’s emotional stance, learners were able to recognize the emotion of the instructor in a video lesson on binomial probability. Specifically, learners generally were able to distinguish positive emotions from negative emotions and had a harder time distinguishing active emotions from passive emotions. Second, concerning social partnership, learners rated the instructor displaying positive emotions as better able to facilitate learning, more credible, and more engaging than the instructor displaying negative emotions. There was not as much support for the third step. There was evidence to show that students reported higher levels of motivation to pay attention to lessons taught by the instructor displaying positive emotions compared to when she displayed negative emotions. But, there were no differences for the remaining questions. Lastly, when we come to the fourth link, having a positive instructor did not lead to better learning outcomes on an immediate test.

Experiment 1 provided support for the initial steps in the positivity principles, and the cognitive affective model of e-learning from which it is derived. In short, the cognitive affective model of e-learning seemed to work up until the last two steps. Although this may show that an instructor’s emotions do not influence perceptions of student effort and learning outcomes, we suspect that it is more likely that this was an assessment problem rather than emotions truly not having an effect on learning. Several major effects on learning—such as the testing effect—do not appear on immediate tests but do appear on delayed tests (Brown et al., 2014; Dunlosky et al., 2013). In addition, learning theorists have long held that deep understanding is better measured by delayed rather immediate tests (Mayer, 2011; Wertheimer, 1959). The next study overcomes this potential assessment problem by employing a delayed test.

## Experiment 2

Experiment 2 is a replication of Experiment 1 with one key difference; participants waited a week between the learning phase and the testing phase. This allows us to determine if the null findings concerning learning outcome from Experiment 1 is an accurate assessment or was caused by an assessment problem. The predictions for this experiment are exactly the same as Experiment 1.

### Method

#### Participants and design

The participants were 114 participants recruited from a psychology subject pool at a university in Southern California. Their mean age was 19.21 years (*SD* = 1.28) and 73 were women, 40 were men, and 1 person did not indicate a gender. The experiment used a 2 (valence of emotion: positive or negative) × 2 (activity: active or passive) design. This created 4 groups; 28 participants were in the happy (positive/active) group, 27 participants were in the content (positive/passive) group, 29 participants were in the frustrated (negative/active) group, and 30 participants were in the bored (negative/passive) group.

#### Materials and apparatus

The materials and apparatus were the same from Experiment 1. The Cronbach’s alpha for prior knowledge items was .72. Cronbach’s alpha for the posttest was .78.

#### Procedure

The procedure was the same as in Experiment 1 except the posttest and post-questionnaire were administered one week after the first session.

### Results

#### Do the groups differ on basic characteristics?

A preliminary issue concerns whether random assignment produced groups that were equivalent on basic characteristics. Concerning prior knowledge score, there were no statistically significant differences between the groups based on valence, *F*(1, 110) = 875.94, *p* = .057, nor based on arousal, *F*(1, 110) = 3.70, *p* = .452, and no significant interaction, *F*(1, 110) = 2.01, *p* = .159. Concerning age, there were no statistically significant differences between the groups based on valence, *F*(1, 110) = .24, *p* = .623, nor based on arousal, *F*(1,110) = .11, *p* = .744, and no significant interaction, *F*(1, 110) = 1.67, *p* = .199. Concerning number of prior math classes taken, there were no statistically significant differences between the groups based on valence, *F*(1, 110) = .13, *p* = .718, nor based on arousal, *F*(1, 110) = .11, *p* = .741, and no interaction, *F*(1, 110) = .85*, p* = .358. Concerning gender, a chi-square test showed that there were no statistically significant differences between the groups, χ^2^(3, *N* = 113) = 5.95, *p* = .114. Based on the fact that there are no significant differences among the groups on any of the statistical tests, we can conclude that participants in each condition were equivalent in the basic characteristics of prior knowledge, age, number of prior math courses, and gender composition.

#### Hypothesis 1: Do students recognize whether an instructor is displaying positive or negative emotions?

The first step in the cognitive affective model of e-learning is to understand if participants are able to recognize the emotions being displayed by the instructor. To address this issue, the data were organized into four sets, one for each emotion (happy, content, ﻿﻿frustrated, and bored). Rating means and standard deviations are reported in Table 5.

**Table 5:** Means and standard deviations of emotional ratings of the 4 video lessons in Experiment 2

	Happy rating		Content rating		Bored rating		Frustrated rating	
	*M*	*SD*	*M*	*SD*	*M*	*SD*	*M*	*SD*
Happy video	**4.18**	.82	4.18	.82	1.79*	1.03	1.36*	.68
Content video	3.67	.78	**3.63**	.88	2.52*	1.05	1.67*	.83
Bored video	1.30*	.84	1.63*	1.00	**4.50**	.97	3.80	1.21
Frustrated video	1.79*	1.21	2.00*	1.34	4.10	1.47	**3.66**	1.65

Asterisk(*) represents significant difference from the target emotion

First, concerning ratings of how happy the instructor was, a 2 (valence: positive, negative) × 2 (activity: active, passive) ANOVA was performed on the ratings of happy emotion that participants reported for the instructor. There was a significant effect of valence favoring the positivity principle, *F*(1, 110) = 185.70, *p* < .001, *d* = 2.52, in which participants who saw the instructor displaying positive emotions (*M* = 3.93, *SD* = .84) gave a higher happy rating than those who saw the instructor displaying negative emotions (*M* = 1.54, *SD* = 1.06). There also was a significant effect of activity, *F*(1, 110) = 8.31, *p* = .005, *d* = .33, in which participants who saw the instructor displaying active emotions (*M* = 2.96, *SD* = 1.58) gave a higher happy rating than those who saw the instructor displaying passive emotions (*M* = 2.42, *SD* = 1.44). There was no significant interaction, *F*(1, 110) = .003, *p* = .957. As a follow-up, we conducted a one-way ANOVA that showed a significant difference in ratings of happy emotion among conditions, *F*(3, 110) = 65.15, *p* < .001. Dunnett’s test (with *p* < .05) revealed that participants rated the instructor in the happy video as significantly happier than both the bored and frustrated videos, but not significantly different from the content video. We conclude that participants were able to recognize that the instructor had a positive emotion when compared to the negative emotions, but had a little harder time distinguishing active from passive emotions. Consistent with hypothesis 1a of the positivity principle, learners were able to tell the difference between the level of happiness when the instructor displayed positive versus negative emotions.

Second, concerning ratings of how content the instructor was, a 2 (valence: positive, negative) × 2 (activity: active, passive) ANOVA was performed on the ratings of content emotion that participants reported for the instructor. There was a significant effect of valence favoring the positivity principle, *F*(1, 110) = 116.21, *p* < .001, *d* = .92, in which participants who saw the instructor displaying positive emotions (*M* = 3.01, *SD* = .89) gave a higher content rating than those who saw the instructor displaying negative emotions (*M* = 1.81, *SD* = 1.18). There was also a significant effect of activity, F = 5.59, *p* = .020, *d* = .33, in which participants who saw the instructor displaying active emotions (*M* = 3.07, *SD* = 1.56) gave a higher content rating than participants who saw the instructor displaying passive emotions (*M* = 2.58, *SD* = 1.37). There was no significant interaction, F = .22, *p* = .639. As a follow-up, a one-way ANOVA was run for the rating of content emotion by each group. There was a significant difference in ratings of content emotion among conditions, *F*(3, 110) = 40.96, *p* < .001. Dunnett’s post-hoc test (at p < .05) revealed that participants in the content group rated the content video as significantly more content than both the bored and frustrated videos, but not significantly different from the happy video. Consistent with hypothesis 1b and the positivity principle, participants were able to recognize that the instructor had a positive emotion as compared to a negative emotion.

Third, concerning ratings of how bored the instructor was, a 2 (valence: positive, negative) × 2 (activity: active, passive) ANOVA was performed on the ratings of bored emotion that participants reported for the instructor. There was a significant main effect favoring the positivity principle, *F*(1, 110) = 99.31, *p* < .001, *d* = 1.85, in which participants who saw the instructor displaying negative emotions (*M* = 4.31, *SD* = 1.25) gave a higher bored rating than those who saw the instructor displaying positive emotions (*M* = 2.15, *SD* = 1.10). Additionally, there was a significant main effect of activity in which participants who saw the instructor displaying passive emotions, *F*(1, 110) = 6.85, *p* = .010, *d* = 2.09, (*M* = 3.56, *SD* = 1.41) gave a higher bored rating than those who saw the instructor displaying active emotions, (*M* = 2.96, *SD* = 1.72). There was no significant interaction, *F*(1, 110) = .61, *p* = .437. A follow-up one-way ANOVA was run for the rating of bored emotion by each group. There was a significant difference in ratings of boredom among conditions, *F*(3, 110) = 35.87, *p* < .001. Dunnett’s post-hoc tests (at *p* < .05) revealed that participants who received the bored instructor rated the video as significantly more bored than those who saw the happy or content instructor, but not significantly different from those who saw the frustrated instructor. Consistent with hypothesis 1c for the positivity principle, participants were able to recognize that the instructor had a negative emotion as compared to a positive emotion.

Lastly, concerning ratings of how frustrated the instructor was, a 2 (valence: positive, negative) × 2 (activity: active, passive) ANOVA was performed on the ratings of frustrated emotion that participants reported for the instructor. There was a main effect of valence in favor with the positivity principle, *F*(1, 110) = 102.60, *p* < .001, *d* = 2.00, in which participants who saw the instructor displaying negative emotions (*M* = 3.73, *SD* = 1.44) gave higher frustrated ratings than those who saw the instructor displaying positive emotions (*M* = 1.51*, SD* = .77). There was no significant main effect of activity*, F*(1, 110) = 1.08*, p* = .301, nor an interaction*, F*(1, 110) = .14*, p* = .707. As a follow-up, a one-way ANOVA was run for the rating of frustrated emotion by each group. There was a significant difference in ratings of frustrated emotion among conditions, *F*(1, 110) = 34.73, *p* < .001. Dunnett’s post-hoc tests (with *p* < .05) revealed that participants in the frustrated group rated the instructor as significantly more frustrated than those in the happy and content groups, but not significantly different from the bored group. Consistent with hypothesis 1d of the positivity principle, participants were able to recognize that the instructor had a negative emotion compared to a positive emotion.

Experiment 2 once again demonstrated that participants are able to recognize whether an instructor is displaying positive or negative emotion. This pattern of results in consistent with step 1 of the cognitive affective model of e-learning.

#### Hypothesis 2: Do students feel more social connection with positive instructors?

As in Experiment 1, the next step in the cognitive affective model of e-learning, shown in Fig. 3, is that once the learner recognized the emotion of the instructor, this should influence the learner’s perception of the instructor as a valued social partner. To assess this idea, the participants gave ratings about three features of the instructor based on the API (Baylor & Ryu, 2003). Means and standard deviations of each factor in the API are reported in Table 6.

**Table 6:** Means and standard deviations for three subscales of the API in Experiment 2

API factors	Positive/active (happy)		Positive/passive (content)		Negative/passive (bored)		Negative/active (frustrated)	
	*M*	*SD*	*M*	*SD*	*M*	*SD*	*M*	*SD*
Facilitating learning	3.11	.80	2.96	.83	1.67	.65	1.90	.94
Credible	3.86	.61	3.61	.59	2.71	.81	2.90	.91
Engaging	3.87	.70	2.90	.85	1.47	.51	1.81	.91

The first factor was the ability of the instructor to facilitate learning. Consistent with the positivity principle, there was a significant effect of valence, *F*(1, 110) = 66.81, *p* < .001, *d* = 1.54, with participants rating the instructor displaying positive emotions (*M* = 3.04, *SD* = .81) as better at facilitating learning compared to the instructor displaying negative emotions (*M* = 1.79, *SD* = .81). There was not a significant effect of activity, *F*(1, 110) = 1.52, *p* = .220, and there was not an interaction, *F*(1, 110) = .04, *p* = .849. The valence of the instructor’s emotion was important in how participants perceived the instructor’s ability to facilitate learning, consistent with hypothesis 2.

The second factor from the API was the instructor’s credibility. Consistent with the positivity principle, there was a significant effect of valence, *F*(1, 110) = 44.24, *p* < .001, *d* = 1.29, with participants rating the instructor displaying positive emotions (*M* = 3.74, *SD* = .61) as more credible than the instructor displaying negative emotions (*M* = 2.80, *SD* = .86). There was not a significant effect of activity, *F*(1, 110) = 2.40, *p* = .124, nor a significant interaction, *F*(1, 110) = .04, *p* = .837. Again, the valence of the instructor’s emotion was important in how participants perceived the instructor’s credibility, again consistent with hypothesis 2.

Lastly, the third factor from the API was how engaging the instructor was. Consistent with the positivity principle, there was a significant effect of valence, *F*(1, 110) = 152.03, *p* < .001, *d* = 2.10, with the instructor displaying positive emotions (*M* = 3.39, *SD* = .91) being rated as more engaging than the instructor displaying negative emotions (*M* = 1.64, *SD* = .77). There was also a significant effect of activity, *F*(1, 110) = 21.41, *p* < .001, *d* = .58 with the instructor displaying active emotions (*M* = 2.82, *SD* = 1.32) being rated as more engaging than the instructor displaying passive emotions (*M* = 2.15, *SD* = .99). There was also a significant interaction, *F*(1, 110) = 5.15, *p* = .025. The interaction revealed that for when the instructor displayed positive emotions, she was rated as significantly more engaging when also active (happy; *M* = 3.87, *SD* = .70) than when she was also passive (content; *M* = 2.90, *SD* = .85), *t*(53) = 4.66, *p* < .001; and for when the instructor displayed negative emotions, she was rated as similarly engaging when she was also active (frustrated; *M* = 1.81, *SD* = .91) and when she was also passive (bored; *M* = 1.47, *SD* = .51), *t*(43.60) = 1.73, *p* = .090. These results are consistent with hypothesis 2.

Overall, the results are once again consistent with the second step in the positivity principle (hypothesis 2). The instructor when displaying positive emotions was better at facilitating learning, more credible, and more engaging than when she displayed negative emotions. Furthermore, there is minimal support for what could be called the enthusiasm principle, in that the instructor when displaying active emotions was more engaging than when she displayed passive emotions for one of the three ratings. In line with hypothesis 2, the positive or negative emotion of the instructor had an effect on how the learner perceived the instructor as a worthwhile social partner.

#### Hypothesis 3: Do students try harder to learn with positive instructors?

In the cognitive affective model of e-learning, the third step is that a positive instructor prime the learner to work harder to try to learn the material. Means and standard deviations of the postquestionnaire questions involving cognitive processing during learning are reported in Table 7. In order to examine hypothesis 3, 2 × 2 ANOVAs were run on each postquestionnaire question.

**Table 7:** Means and standard deviations for postquestionnaire questions in Experiment 2

Questionnaire items	Positive/active (happy)		Positive/passive (content)		Negative/passive (bored)		Negative/active (frustrated)	
	*M*	*SD*	*M*	*SD*	*M*	*SD*	*M*	*SD*
Pay attention	3.18	1.09	2.96	1.22	1.63	.89	2.00	1.00
Difficulty	2.93	1.09	2.85	1.26	2.93	1.17	3.17	1.34
Effort	2.39	.99	2.89	1.01	2.70	.88	2.48	1.12
Enjoy	2.61	1.23	2.74	1.23	2.13	1.01	1.97	1.21
More lessons	2.39	1.26	2.67	1.52	1.70	.88	1.62	1.08

The first question analyzed was “I was motivated to pay attention to the lesson I just watched.” As in Experiment 1, there was a significant effect of valence, *F*(1, 110) = 40.34, *p* < .001, *d* = 1.20, with participants indicating they were motivated to pay attention more when the instructor displaying positive emotions (*M* = 3.07, *SD* = 1.15) compared to when she displayed negative emotions (*M* = 1.81, *SD* = .95). There was no effect of activity, *F*(1, 110) = 2.17, *p* = .143, nor an interaction, *F*(1, 110) = .15, *p* = .703. Consistent with hypothesis 3 of the positivity principle, participants tried to pay attention more to the instructor when the instructor was positive than when the instructor was negative.

The second question analyzed was “I put a lot of effort to understand the information in the lesson.” There was no effect of valence, *F*(1, 110) = .07, *p* = .793, no effect of activity, *F*(1, 110) = 3.59, *p* = .061, and no significant interaction, *F*(1, 110) = .55, *p* = .461. These findings are not consistent with hypothesis 3.

The third question analyzed was “The information in the lesson was difficult for me.” There was no effect of valence, *F*(1, 110) = .51, *p* = .478, no effect of activity, *F*(1, 110) = .48, *p* = .491, nor an interaction, *F*(1, 110) = .13, *p* = .723, which is not consistent with hypothesis 3.

The fourth question analyzed was “I enjoyed learning about this information.” There was an effect of valence in line with the positivity principle, *F*(1, 110) = 8.12, *p* = .005, *d* = .48, with the instructor displaying positive emotions (*M* = 2.61, *SD* = 1.22) eliciting higher ratings of enjoyment than those elicited when she was displaying negative emotions (*M* = 2.05, *SD* = 1.10). There was no effect of activity, *F*(1, 110) = .47, *p* = .493, nor an interaction, *F*(1, 110) = .01, *p* = .938. Consistent with hypothesis 3 of the positivity principle, participants enjoyed the lesson more if the instructor was positive than if the instructor was negative.

The fifth question analyzed was “I would like more lessons like this one.” There was an effect of valence, *F*(1, 110) = 14.99, *p* < .001, *d* = .67, with the instructor displaying positive emotions (*M* = 2.53, *SD* = 1.39) leading to participants wanting more similar lessons compared to when she displayed negative emotions (*M* = 1.66, *SD* = 1.22). There was no effect of activity, *F*(1, 110) = .62, *p* = .433, nor an interaction, *F*(1, 110) = .19, *p* = .666. Consistent with hypothesis 3 of the positivity principle, participants reported that they would like more lessons like this one when the instructor was positive compared to when the instructor was negative.

Overall, there is limited support based on three of the five subjective self-report measures that the instructor’s emotion influenced the how the participants approached the lesson. In particular, the three questions showing support for this step are the ones assessing more affective characteristics of the learners and the ones not showing support assess more cognitive characteristics.

#### Hypothesis 4: Do students learn better from positive instructors?

The last step in the cognitive affective model of e-learning is that learners who had a positive instructor should perform better on a delayed posttest than learners who had a negative instructor. As explained earlier, there may not have been a difference in the immediate test in Experiment 1 because understanding is not as well measured by an immediate test as by a delayed test. Means and standard deviations of the posttest are reported in Table 8, and as in Experiment 1, a 2 × 2 ANOVA was conducted on the posttest scores. There was a significant effect of valence, *F*(1, 110) = 8.77, *p* = .004, *d* = .54, with the instructor displaying positive emotions (*M* = .51, *SD* = .20) producing higher posttest scores than the instructor displaying negative emotions (*M* = .41, *SD* = .17). There was not a significant effect of activity, *F*(1, 110) = .01, *p* = .927, nor an interaction, *F*(1, 110) = 1.18, *p* = .281. These results are consistent with hypothesis 4 and with a major prediction of the positivity principle. The valence of the instructor’s emotion affected how well participants performed on a delayed posttest.

**Table 8:** Means and standard deviations for posttest in Experiment 2

	Positive/active (happy)		Positive/passive (content)		Negative/passive (bored)		Negative/active (frustrated)	
	*M*	*SD*	*M*	*SD*	*M*	*SD*	*M*	*SD*
Post-test	.49	.18	.53	.22	.43	.18	.39	.15

### Discussion

As in Experiment 1, Experiment 2 demonstrated that the cognitive affective model of e-learning applies to a video lecture. In Experiment 2, there was evidence for each of the four steps in the positivity principle and the cognitive affective model of e-learning from which it is derived. First, learners are able to distinguish between the emotions of an instructor, particularly whether the emotional tone was positive or negative. Second, participants see the instructor as better at facilitating learning, more credible, and more engaging when the instructor is positive compared to when the instructor is negative. Third, learners pay more attention to and like positive instructors more than negative ones, although participants still did not report differences in cognitive characteristics. Finally, in contrast to Experiment 1, learners perform better on delayed posttests if they had positive rather than negative instructors.

This study not only demonstrates how the cognitive affective model of e-learning can work in an online instructional video lecture, but it also demonstrates how delayed tests are important for testing understanding. In short, Experiment 2 helped confirm that the emotion of the instructor has an influence on learning, in which positive instructors led to better learning processes and outcomes than negative instructors.

## General discussion

### Empirical contributions

The main goal of this study was to examine four main findings keyed to four hypotheses concerning learning from positive instructors (i.e., displaying happy or content emotion) versus negative instructors (i.e., displaying bored or frustrated emotion). First, learners were able to recognize the emotional tone of the instructor in an instructional video lecture, particularly whether then instructor was exhibiting positive or negative emotions in Experiments 1 and 2. This is reflected in participants giving higher happy and content ratings for the happy and content instructors, and higher frustrated and bored ratings to the frustrated and bored instructors. Second, learners rated a positive instructor (i.e., happy or content instructor) as more likely to facilitate learning, more credible, and more engaging than a negative instructor (i.e., frustrated or bored instructor) in Experiments 1 and 2. Third, learners reported paying more attention during learning for a positive instructor than a negative instructor in Experiments 1 and 2, and the delayed test (in Experiment 2) showed additional differences in assessing the participants' affective impressions of the lecture. Finally, learners who had a positive instructor scored higher than learners who had a negative instructor on a delayed posttest (in Experiment 2) but not an immediate posttest (in Experiment 1). In short, students were better able to answer statistics problems (on a delayed test but not an immediate test) after viewing a statistics lesson delivered by a positive instructor than a negative instructor. Overall, there is evidence for the positivity principle, which states that people respond to and learn better from positive instructors than from negative instructors.

### Theoretical implications

The pattern of results is at least partially consistent with each of the four links in the cognitive affective model of e-learning summarized in Fig. 3, in which learners recognize whether an instructor is exhibiting positive or negative emotional tone, feel more social connection with a positive instructor than a negative one, engage more deeply in learning from a positive instructor than a negative one, and perform better on a delayed learning outcome posttest after learning with a positive instructor than a negative one.

This study also shows the usefulness of Russell’s (1980, 2003) model of core affect within the context of e-learning with instructional video lectures. In line with Russell’s model of core affect, learners in the present experiments were sensitive to the emotional tone of the instructor in terms of whether the instructor displayed positive emotions (i.e., happy or content) or negative emotions (i.e., frustrated or bored) and to a lesser extent in terms of whether the instructor displayed active emotions (i.e., happy or frustrated) or passive emotions (i.e., content or bored).

Overall, this work demonstrates the need to incorporate affective factors into cognitive learning theories such as the cognitive theory of multimedia learning (Mayer, 2020a, in press) and cognitive load theory (Paas & Sweller, 2014; Sweller et al., 2011), which focus mainly on cognitive processes in learning. The cognitive affective model of e-learning multimedia provides a basic framework for how to combine affective and cognitive features, but more work is needed to build a detailed account, perhaps building on the cognitive-affective theory of learning with media (Moreno & Mayer, 2007) and the integrated cognitive affective model of learning with multimedia (Plass & Kaplan, 2016). Additionally, these findings provide support for the idea that a positive emotional tone of an instructor benefits learning due to its ability to motivate students to pay attention to the lesson at a higher intensity than a negative emotional tone of an instructor (Plass & Kalyuga, 2019).

### Practical implications

This project offers practical implications for how to design instructional video presented on a computer screen. The most straightforward instructional design principle suggested by this study is that instructional designers should be aware of the emotional tone displayed by instructors in computer-based lessons involving instructional video. This study demonstrates the power that emotions displayed by instructors, especially positive vs negative emotions, have on learning with instructional videos. It follows that instructors in video lectures should be aware of the emotional tone they take while teaching and its potential effects on students. This study was conducted using video lectures to be displayed on computer screens (such as a resource in a learning management system or in MOOCs), so it follows that instructors creating video lectures should be aware of the emotional stance they take in presenting the material.

Specifically, this work suggests that instructors in computer-based instructional video should display a positive emotional tone while lecturing. Positive emotion—particularly a happy or content emotion—is conveyed through voice, body stance, gesture, facial expression, and eye-gaze. More work is needed to offer specific prescriptions for specific types of learners and learning situations. Designing lessons with positive emotion applies to computer-based learning from instructional video, because even in a video lecture, the emotional tone of the instructor affects learning processes and outcomes. Displaying positive emotion in video lectures can be seen as a form of emotional design (Loderer et al., 2020; Mayer & Estrella, 2014; Mayer, 2020b; Plass & Kaplan, 2016; Plass et al., 2014; Um et al., 2012).

As prerecorded video lectures are becoming more commonplace, due to COVID-19 and interest in virtual education, it is vital to understand how this technology will have an impact on learners. This research demonstrates how learners can read the emotion of an instructor from a video lesson and how that impacts their learning of that material. So, for those interested in using this type of technology, it is vital they are aware of the cognitive impacts of affect.

### Methodological implications

A major methodological implication of this study is that assessments of instructional effectiveness should be conducted with delayed tests in addition to immediate tests. Although the effects of an instructor’s positive emotions did not appear on an immediate test (in Experiment 1), they did appear on a delayed test (in Experiment 2). We do not recommend asking the same learners to take both an immediate and delayed test because the act of taking an immediate test is itself a learning episode that can influence performance on the delayed test. To avoid this kind of testing effect (Brown et al., 2014; Dunlosky et al., 2013), we recommend giving immediate and delayed tests to different learners as was done in this set of experiments.

### Limitations and future directions

There were a few limitations to these studies that should be noted. First, the video presented a lesson on statistics. Participants’ interest in statistics can be varied, and thus, some may have had certain attitudes towards the lesson that had an effect on their perception of the instructor’s affect or on the lesson itself. Future research should investigate how the emotional tone of an instructor plays a role in learning in lessons outside of statistics, as the instructor’s emotional tone may be less relevant when the lesson itself excites the learners.

Additionally, this study involved a short lesson, which lasted only about 10 min. This does not mimic how learning typically occurs in a classroom setting or in online courses. In classroom settings and online courses, material often builds off previously learned information and requires learners to integrate their knowledge between the lessons, which was not the case in the present study. Furthermore, the emotional impact of an instructor over time may also play a diminishing role in how much learners gain from multiple lessons. Future research should investigate how the emotional tone of an instructor impacts learning over a longer term and in both classroom contexts and online courses.

One of the steps of our hypothesis was not fully supported in this study when using metacognitive self-report measures. Although this could indicate that step 3 may not have a strong impact on learning, the results could also be due to the problems students have with self-reports about metacognition. One potential problem is that participants may not answer the self-report accurately, either due to failure to be aware of their internal experience or due to wanting to please the experimenters with their responses. Future research should investigate step 3 of this hypothesis more carefully by investigating the effort someone puts into learning a lesson in a way that doesn’t involve self-report. In addition, future research should investigate the impact of asking for the self-ratings on an immediate post-questionnaire rather than after a delay.

This study was conducted with students at a U.S. university, and the U.S. is a highly diverse place. Emotions and how emotions are displayed vary culturally, meaning that the results of these studies may not generalize to a larger population (e.g., Engelmann & Pogosyan, 2013; Fang et al., 2017; Grossmann et al., 2011). Because different cultures view emotion and the display of emotions differently, it may be the case that learners from different cultures would react to the emotional tone of an instructor differently. Future research should investigate how culture may play a role in how learners respond to the emotional tone of an instructor.

Furthermore, this research appears to contradict some prior findings in which inducing positive emotions in learners prior to a lesson negatively affects learning (e.g., Knörzer et al., 2016). One difference between the current study and this previous one is in the previous study, emotions were induced prior to the learning and were unrelated to the lesson itself, influencing the way a learner enters a learning experience. In contrast, in the present study, the emotions were more closely tied to the content of the lesson because we focused on how an instructor’s emotion while teaching a lesson influenced learning. This difference may be a reason why these studies found different impacts; coming into a lesson with an already established positive emotion may cause the emotion to be more of a cognitive distraction during learning, whereas the positive emotion an instructor uses to explain material may become more of a motivator for the learner to become engaged with the material. Further work should continue to investigate the educational implications of this distinction between inducing emotion before or within the context of learning.

Future research should investigate how changing the instructor impacts learners. We only had one instructor for all the videos, a young woman. Similarly to the point above, changing who is instructing the students could change how the emotions impact the learner.

Future research should investigate the arousal (or what we call the activity) dimension of Russell’s (1980, 2003) model of core affect. In this study, participants had a harder time distinguishing between an active instructor and a passive instructor for both positive and negative emotions. This difficulty may have been due to our actress not being able to portray each type of arousal (or activity) distinctly. Alternatively, it may be the case that participants are less sensitive to the active–passive dimension of emotion and focus mostly on the positive–negative dimension of the emotion.

## Conclusion

This research represents an attempt to understand what role an instructor’s displayed emotion plays in student learning. We proposed the cognitive-affective model of e-learning that include 4 links that occur when an instructor displays an emotion during instruction: (1) the learner perceives the instructor’s emotion, (2) the learner builds a social partnership with the instructor, (3) the learner exerts more effort to understand the material in the lesson, and (4) the learner builds a better learning outcome. These two experiments demonstrate that learners can recognize the valence (positive vs negative) of an instructor’s emotion (link 1), which then influences how the learner views that instructor as a social partner (link 2). This then influences the motivation of the learner (link 3) and benefits understanding of the material as indicated on a delayed test but not an immediate test (link 4).

## Appendix A

### Script from lesson

Hi everyone. Imagine that you are trying to impress your friends with your ability to predict what will happen if you roll a die a certain number of times. For example, suppose you win if you roll 5 or 6 and you lose if you roll 1, 2, 3, or 4. Let's say you roll the die 5 times and you win 2 times and lose 3 times. What exactly is the probability of that happening? Today, I will help you understand how to answer questions like this one. This is called binomial probability.

First, you need to understand trials and outcomes. A trial is something you do. For example, you roll a die. An outcome is what happens on the trial. For instance, if you roll a die (the trial), the outcome could be that you rolled a 4.

Second, you also need to think about success and failure. A success is defined, by you, as one or more of the possible outcomes. For example, a success of rolling the die could be that you roll a number greater than 4. That means, if you roll a die, and get a 5 or 6, a success has occurred. On the other hand, a failure occurs on any trial that is not a success. So, if you defined success as rolling a number greater than 4, failure would occur if you rolled a 1, 2, 3, or 4.

Next, we should figure out the probability of success. The probability of success is the number of success outcomes divided by the total number of outcomes (including the success outcomes) if all the outcomes have an equal chance. In this case, there are 6 equally likely outcomes and 2 of them are successes, so the probability of success is 2 out of 6 or one-third. We can expect a 5 or a 6 to come up on about one-third of the times the die is rolled. The probability of success can be symbolized by the letter P.

Similarly, there is a probability of failure. This is the probability of success subtracted from 1. So, in our example, the probability of failure is 1 minus one-third which is two-thirds. The probability of failure can be symbolized as 1 minus P.

Now you know how to determine the probability of success (symbolized as P) and the probability of failure (symbolized as 1 minus P).

The next concept you need to know is sequence. A sequence is what happens when you conduct several trials, one after another, like rolling a die 5 times in a row. For each trial, we have either a success or a failure, so the sequence reports what occurred. For example, say we rolled a die 5 times in a row and rolled a 2, then a 4, then a 6, then a 2, and then a 5. The sequence would be failure, failure, success, failure, success.

A sequence, like the previous example, has a probability of occurring, which is called the joint probability of a sequence. This can be found by multiplying the probabilities of each individual event. Let’s take the previous example. We had failure, failure, success, failure, success. Now, we multiply the probability of each happening, so we get two-thirds (for failure), times two-thirds (for failure), times one-third (for success), times two-thirds (for failure), times one-third (for success). We can also write this as one-third squared times two-thirds cubed. So, the joint probability of this particular sequence occurring is 8 out of 243.

We can compute the probability for any specific sequence. So, let's say the number of trials in a sequence can be symbolized by the letter N and the number of successes in those trials is called R and the number of failures is N minus R. To figure out the probability of any sequence, you can use the formula displayed on the screen. We multiply the probability of success (P) by itself R times, then we multiply the probability of failure (1 minus P) by itself N minus R times, and we finally multiply those two numbers together. This is called the joint probability of a sequence.

Now you know how to compute the joint probability of a sequence of successes and failures. The next step is to figure out how many different sequences (that is, patterns of successes and failures) have that same number of successes out of N trials. For example, there are three different ways that we can have 2 successes from 3 trials:

success, success, failure

success, failure, success

failure, success, success

As you can see, in each sequence, there are 2 successes and 1 failure. The number of different sequences having R successes in N trials is called the number of combinations. In this example, there are 3 combinations for a sequence having 2 successes out of 3 trials.

The number of combinations may be simple to work out by hand when there are just a few trials, like our previous example, but what if I asked you how many different combinations can occur for 2 successes in 5 trials? In cases like this, having a formula to find the number of combinations is quite helpful. This formula is N factorial divided by R factorial times N minus R factorial. This equation includes a factorial symbol (indicated by an exclamation point). This factorial symbol means multiply the number before the exclamation mark times the number minus one, then times the number minus two, and so on down to 1. For example, 5 factorial equals 5 times 4 times 3 times 2 times 1, which equals 120.

Now, let’s finish finding the number of combinations that can occur for 2 successes in 5 trials. So, 5 factorial is equal to 120, which we just found out. Then, we divided that by 2 factorial (which is 2 times 1) times 5–2 factorial, or 3 factorial (which is 3 times 2 times 1). That gives us 120 divided by 12, which equals 10. This means there are 10 ways to get 2 successes in 5 trials.

Now you see how to compute the joint probability of a particular sequence that has R successes in N trials (such as failure, failure, success, failure, success) and how to compute the number of combinations in which a sequence has R successes in N trials (such as 10 ways to get 2 successes out of 5 trials).

As the final step in computing binomial probability you just put those two parts together. You can figure out the probability of getting R successes out of N trials by multiplying the number of combinations for a sequence that has R successes out of N trials by the joint probability of any one of those sequences. When you do this, you are finding the probability of R successes in N trials. So, if you put that all together you get the formula on the screen. This is what we call a binomial probability.
